# Expression and subcellular localization of Discoidin Domain Receptor 1 (DDR1) define prostate cancer aggressiveness

**DOI:** 10.1186/s12935-021-02206-1

**Published:** 2021-09-21

**Authors:** R. Daniel Bonfil, Wei Chen, Semir Vranic, Anjum Sohail, Dongping Shi, Hyejeong Jang, Hyeong-Reh Kim, Marco Prunotto, Rafael Fridman

**Affiliations:** 1 Division of Pathology, Dr. Kiran C. Patel College of Allopathic Medicine, Nova Southeastern University, 3200 S. University Drive, Terry Building # 1337, Fort Lauderdale, FL 33328-2018 USA; 2 Department of Oncology, Wayne State University School of Medicine and Karmanos Cancer Institute, Detroit, MI USA; 3 Department of Pathology, Wayne State University School of Medicine and Karmanos Cancer Institute, Scott Hall #8200, 540 E. Canfield St, Detroit, MI 48201 USA; 4 College of Medicine, QU Health, Qatar University, Doha, Qatar; 5 Biomedical and Pharmaceutical Research Unit, QU Health, Qatar University, Doha, Qatar; 6 School of Pharmaceutical Sciences, Institute of Pharmaceutical Sciences of Western Switzerland, University of Geneva, Geneva, Switzerland

**Keywords:** DDR1, Prostate cancer, Receptor tyrosine kinases, Immunohistochemistry, Prognostic markers

## Abstract

**Background:**

The Discoidin Domain Receptor 1 (DDR1) is one of the two members of a unique family of receptor tyrosine kinase receptors that signal in response to collagen, which has been implicated in cancer progression. Here, we examined the expression of DDR1 in prostate cancer (PCa), and assessed its potential value as a prognostic marker, as a function of grade, stage and other clinicopathologic parameters.

**Methods:**

We investigated the association between the expression level and subcellular localization of DDR1 protein and PCa aggressiveness by immunohistochemistry, using tissue microarrays (TMAs) encompassing 200 cases of PCa with various Gleason scores (GS) and pathologic stages with matched normal tissue, and a highly specific monoclonal antibody.

**Results:**

DDR1 was found to be localized in the membrane, cytoplasm, and nuclear compartments of both normal and cancerous prostate epithelial cells. Analyses of DDR1 expression in low GS (≤ 7[3 + 4]) vs high GS (≥ 7[4 + 3]) tissues showed no differences in nuclear or cytoplasmic DDR1in either cancerous or adjacent normal tissue cores. However, relative to normal-matched tissue, the percentage of cases with higher membranous DDR1 expression was significantly lower in high vs. low GS cancers. Although nuclear localization of DDR1 was consistently detected in our tissue samples and also in cultured human PCa and normal prostate-derived cell lines, its presence in that site could not be associated with disease aggressiveness. No associations between DDR1 expression and overall survival or biochemical recurrence were found in this cohort of patients.

**Conclusion:**

The data obtained through multivariate logistic regression model analysis suggest that the level of membranous DDR1 expression status may represent a potential biomarker of utility for better determination of PCa aggressiveness.

**Supplementary Information:**

The online version contains supplementary material available at 10.1186/s12935-021-02206-1.

## Introduction

The Discoidin Domain Receptors (DDRs) belong to the family of receptor tyrosine kinases (RTKs) that signal in response to collagen, the major component of extracellular matrices. Whereas the majority of the ligands for RTKs are soluble diffusible proteins, the DDRs are the only receptors that recognize collagens as ligands [1, 2]. Upon binding to collagen, DDRs undergo phosphorylation in their intracellular domains [3, 4], which activate downstream effectors to initiate signaling pathways that control cell fate and behavior. Thus, DDRs are major mediators of cell–matrix interactions.

The DDR family is comprised of two members, DDR1 and DDR2, which are encoded by two distinct genes located in different chromosomes [2]. While there is only one DDR2 form, the DDR1 subfamily of receptors is comprised of five isoforms generated by alternative splicing from which three are fully functional kinases (DDR1a, DDR1b, and DDR1c), and two are truncated receptors (DDR1d and DDR1e) lacking enzymatic activity. At present, the cellular distribution and functional roles of each DDR1 isoform in different tissues remain unclear. DDR1 is usually expressed by epithelium whereas DDR2 is mostly expressed by mesenchymal cells. However, this distinction is not clearly maintained during pathological conditions [1, 5]. Structurally, both DDRs are type I transmembrane proteins and have a characteristic N-terminal extracellular domain that is homologous to the *Dictyostelium discoideum* *N*-acetylgalactosamine-binding lectin Discoidin-I, referred to as Discoidin domain (DS), which mediates binding to collagens. The DS domain is followed by a DS-like domain, an extracellular juxtamembrane region, a transmembrane region, and an intracellular cytoplasmic region that contains the kinase domain at the C-terminus [2, 6]. DDRs undergo receptor activation by both non-fibrillar and fibrillar collagens as their ligands, DDR1 recognizing both types of collagens, whereas DDR2 mostly fibrillar collagens. DDR1 is uniquely activated by collagen IV, and thus DDR1 can mediate the interactions of epithelial cells with the basement membrane. Another feature that sets the DDRs apart from other RTKs is their delayed and long-lasting phosphorylation upon binding to their ligands [2, 7].

An increasing number of studies suggest a pivotal role of DDRs in cancer progression [5, 8–10]. Levels of DDRs are usually elevated in a variety of cancer types, which have been associated with malignant progression and poor patient survival [5, 8, 10–14]. Consistently, findings in experimental models of cancer have shown that DDRs play a key role in tumorigenesis and metastatic ability [15–23]. Therefore, DDRs are considered promising therapeutic targets in various cancer types, and several small molecules kinase inhibitors of DDRs have been developed [24–26].

Prostate cancer (PCa) is the most commonly diagnosed non-skin malignant neoplasia in males in the United States [27]. Despite the management of the disease when still confined to the gland with local therapies such as radical prostatectomy (RP), endocrine, and radiation therapy (RT), not all patients can be definitively cured. In fact, 20–40% of patients treated with RP [28, 29] and 30–50% of those treated with RT [30] experience rising serum prostate-specific antigen (PSA) levels—a condition known as biochemical recurrence (BCR)—within 10 years. Although progression to metastases occurs in some patients after BCR, the latter is not considered a surrogate for metastatic disease or PCa-specific mortality [31, 32]. Conversely, Gleason score (GS) remains one of the strongest pathologic indicators of PCa aggressiveness, with a GS of 6 or below indicating a lower risk of PCa-specific mortality and a GS of 8 or above indicating a higher risk [33]. However, prognosis prediction in patients with GS 7 prostate tumors, designed as “intermediate risk PCas”, is challenging due to a marked heterogeneity of clinical outcomes that may result from an actual difference in aggressiveness between Gleason 3 + 4 = 7 and 4 + 3 = 7 tumors [34–36], now referred to as Grades Group 2 and 3, respectively [37, 38]. Therefore, the identification of novel molecular markers that may improve risk stratification and lead to novel therapies for certain PCa patients is critically needed. A few studies examined the expression of DDR1 [39] and DDR2 [40] in PCa tissues. DDR1 protein was found to be expressed in PCa clinical specimens and correlated with androgen-independence, but not associated with GS score or PSA levels [39]. High DDR2 expression in advanced PCa was found to strongly correlate with PSA, GS, and lymph node metastasis [40]. In the present study, we utilized a highly specific and validated monoclonal antibody to human DDR1 that was specifically developed for immunohistochemistry (IHC) analyses [41, 42]. Using this antibody, we evaluated the expression of DDR1 in various subcellular fractions (membrane, cytoplasm, and nucleus) in a cohort of PCa samples with tumor and adjacent normal epithelium that, to our knowledge, is the largest set of PCa specimens studied for this RTK up to date. Specifically, we assessed the association between DDR1 expression and subcellular localization and PCa aggressiveness, and its potential value as a prognostic marker, as a function of grade, stage and other clinicopathologic parameters.

## Materials and methods

### Prostate tissue specimens

The Grade/Stage tissue microarray (TMA) slides containing de-identified human PCa specimens were obtained from the Prostate Cancer Biorepository Network (PCBN), a public bioresource for PCa investigators funded by the Department of Defense resulting from a collaboration between Johns Hopkins University and New York University School of Medicine (Provider Investigator: Dr. Bruce Trock, Johns Hopkins University, Baltimore, MD). Five TMAs referred to as TMA 18, 19, 22, 24, and 34 containing 1,600 cores obtained from radical prostatectomies performed in PCa patients were analyzed in our study. The TMAs comprise a total of 200 cases of PCa with various GS and pathology stages represented in quadruplicate cores, and matching non-cancerous prostate tissues referred to as “normal” (also 4 cores per case). All subjects were annotated based on age, race, treatments received after radical prostatectomy, biopsy GS, serum PSA levels at diagnosis, clinical stage (T) as defined by the American Joint Committee on Cancer (Table 1), and occurrence of BCR (increase of postoperative serum PSA level to 0.2 ng/ml) (Additional file 1: Table S1). None of the patients received preoperative therapy (chemo-, radio- or hormonal therapy).

**Table 1:** Demographics and clinicopathologic features associated with TMA cohort

Variable	Median	Range
Age at diagnosis (years)	57	36–73
Body mass index	26.54	20.04–39.15
PSA at diagnosis (ng/ml)	5.99	1.29–38

Variable	Frequency	%
Race		
African American	17	8.5
Asian	2	1
Caucasian	173	86.5
Other	8	4
Family history of prostate cancer		
No	112	56
Yes	73	36.5
TNM stage		
Local	132	66
Advanced	68	34
Gleason score		
6	110	55
7 (3 + 4)	56	28
7 (4 + 3)	11	5.5
8	14	7
9–10	9	4.5
Extraprostatic extension		
No	136	68
Yes	63	31.5
Lymph node metastases		
No	195	97.5
Yes	3	1.5
Distant metastasis		
No	165	82.5
Yes	6	3
Adjuvant therapy^a^		
None	169	84.5
Adjuvant radiation only	2	1
Chemotherapy^a^		
No	168	84
Yes	3	1.5
Radiation therapy^a^		
No	160	80
Yes	11	5.5
Hormonal therapy^a^		
No	160	80
Yes	12	6
Surgical margin status		
Negative	169	84.5
Positive	31	15.5
Seminal vesicle involvement		
No	186	93
Yes	14	7

Numbers do not always add up to 200 (or 100%) in some categories because of cases with missing data

^a^All treatment modalities were applied postoperatively

### Immunohistochemistry

TMA sections were deparaffinized with xylene and rehydrated with decreasing percentages of ethanol (100% to 70%). Unmasking of antigenic epitopes was performed with Antigen Retrieval Citrus Plus Solution (Cat. # HK0805K, BioGenex, Fremont, CA) in a pressure cooker placed in a microwave for two cycles of 15 min at heat levels 5 and 2. After 20 min at room temperature, the sections were washed with phosphate-buffered saline (PBS) and endogenous peroxidase quenched with 3% hydrogen peroxide in PBS for 30 min. After washing with PBS and blocking of non-specific binding sites with 2.5% normal horse serum for 20 min at room temperature, the tissues were incubated with an in-house raised rabbit monoclonal antibody (mAb) against the extracellular domain of human DDR1 [41] referred to as Rab‑819 antibody, at a 1:100 dilution (from a stock solution of 1 mg/ml) overnight at 4 °C. Immunostaining was performed by incubation with a peroxidase micropolymers attached to anti-rabbit IgG made in horse (Cat. # MP-7401, ImmPRESS™, Vector Laboratories, Burlingame, CA) for 30 min at room temperature, followed by detection with ImmPACT™ DAB peroxidase substrate (Cat. # SK-4105, Vector Laboratories) and light nuclear counterstaining with Mayer’s hematoxylin (Cat. # HMM500, ScyTek Laboratories, Logan, UT). DDR1 immunoreactivity was evaluated and reported by two independent pathologists (SV and DS) as positive or negative staining in different subcellular localizations (i.e., plasma membrane, nucleus, and cytoplasm) in cancerous and adjacent normal (no evidence of neoplastic changes) tissues. For membrane immunostaining, only full membranous staining was considered positive, whereas no staining or staining at basal or basolateral locations were defined as negative.

### Tissue culture

The human PCa PC-3 and C4-2B cell lines were purchased from the American Type Culture Collection (ATCC). Normal human prostate epithelial cell lines, CF-91, ML-8891, CLR-2221, RWPE-1, RWPE-2, and benign prostatic hyperplasia (BPH) epithelial cell lines were kindly provided by Dr. S. Sheng [Wayne State University (WSU)], whereas human PCa LNCaP and DU145 cell lines were supplied by Dr. H-R.C. Kim (WSU). Normal prostate epithelial cell lines, CF-91, ML-8891, CLR-2221, RWPE-1, RWPE-2 were maintained in Keratinocyte Serum Free Medium (Cat. # 17005042, Thermo Fisher Scientific, Waltham, MA.) supplemented with 0.05 mg/ml bovine pituitary extract and 5 ng/ml epidermal growth factor. PC3, LNCaP and C4-2B cell lines were maintained in RPMI-1640 Medium, HEPES (Cat. # 22400121 Thermo Fisher Scientific), supplemented with 10% Fetal Bovine Serum (FBS) (Cat. # 16000044, Thermo Fisher Scientific). The DU145 cell line was cultured in DMEM Medium (Cat. # 10313039, Thermo Fisher Scientific) supplemented with 10% FBS and 1% Penicillin–Streptomycin solution. All the human cell lines used were periodically confirmed negative for mycoplasma contamination and authenticated through short tandem repeat profiling by the Research Technology Support Facility of Michigan State University.

### Immunoblot analyses, collagen stimulation, and cell fractionation

For analyses of DDR1 expression in whole cell lysates, cultured prostate epithelial cells were lysed in RIPA buffer (50 mM Tris–HCl, pH 7.4, 150 mM NaCl, 1% NP-40, 0.25% sodium deoxycholate and 1 mM EDTA) supplemented with protease inhibitors Cocktail, EDTA-free (Cat. # 539134, Sigma-Aldrich, St. Louis, MO) and 10 mM sodium fluoride and 1 mM sodium orthovanadate. The cell lysates were cleared by centrifugation at 13,000*g* at 4 °C for 15 min, and the protein concentration was determined using the BCA kit from Pierce (Cat. # 23227, Waltham, MA). Equal amounts of protein from each lysate were resolved by reducing 7.5% SDS-PAGE. Proteins were then transferred to a nitrocellulose membrane using conventional methods. The blots were probed with anti-DDR1 polyclonal antibody Sc-532 (Santa Cruz Biotechnology, Inc. Dallas, Texas), which recognizes a DDR1 epitope at the C-terminal end of the receptor. For loading control, the same blot was reprobed with anti-β-actin antibody.

For analyses of DDR1 subcellular localization in malignant (PC-3) and non-malignant (RWPE-1) cells as a function of collagen stimulation, cells were washed twice with PBS and incubated in serum-free media, overnight. The cells were then treated with 20 µg/ml rat tail collagen type I (Cat. # 354236, Discovery Labware Inc., Corning™, Bedford, MA) for 90 min at 37 °C, washed twice with cold PBS and then gently dissociated from the plates using Cell Dissociation Buffer (Cat. # 13151014, Thermo Scientific). The cytoplasmic/membrane and nuclear fractions were isolated using the NE-PER Nuclear Cytoplasmic Extraction Reagent kit (Cat. # 78833, Thermo Scientific, Grand Island, NY, USA), according to the manufacturer’s instruction. Cytoplasmic and nuclear extraction buffers were supplemented with protease inhibitors (Roche, complete, Mini, EDTA-free) and 10 mM sodium fluoride and 1 mM sodium orthovanadate. Protein concentrations were determined using the BCA kit. For immunoblot analyses, the nuclear and cytoplasmic/membrane fractions were resolved by SDS-PAGE in two gels: 7.5% polyacrylamide for DDR1 and 4–20% polyacrylamide for glyceraldehyde 3-phosphate dehydrogenase (GAPDH) and Histone. After transfer, the first membrane was probed with an antibody recognizing phosphorylated DDR1 at Tyr513, namely DDR1 rabbit mAb E1N8F (Cat. # 14531) from Cell Signalling Technology (CST), Danvers, MA. After stripping, the membranes were re-probed with total anti-DDR1 (D1G6) rabbit mAb (Cat. # 5583, CST), which recognizes the C-terminal region of DDR1. The membrane was also reprobed for presence of transferrin receptor as a marker of membrane-anchored protein, using an anti-transferrin mouse mAb (Cat. # 612124) from BD Transduction Laboratories, San Jose, CA. The second membrane was probed with anti-Histone H1 mouse mAb (Cat. # 05-457, Sigma-Aldrich) and re-probed with anti-GAPDH mouse mAb (Cat. # MA5-15738, Thermo Fisher Scientific), as nuclear and cytoplasmic protein markers, respectively. Antigen/antibody complexes were visualized using the SuperSignal West Pico Plus and/or the SuperSignal West Femto Maximum Sensitivity Substrate from Thermo Fisher Scientific (Rockford, IL; Cat. # 34580 and 34095, respectively).

### Statistical methods

The primary objective was to evaluate the association between DDR1 IHC expression and GS, comparing between “low grade” (GS 3 + 4 or lower) and “high grade” (GS 4 + 3 or higher) tumors. For each PCa case, we summarized the quadruple core-level DDR1 data to patient-level data with overall staining percentage (OSP), a semi quantitative score defined as the percentage of positive stained cores among all quadruple cores, per tissue type (cancerous and adjacent normal), and per subcellular location (membrane, nuclear, or cytoplasm). TMA cores that had stroma, no glands, or no tissue after the staining process were considered as missing at random rather than negative staining.

Association between high/low grade GS and dichotomized DDR1 staining OSP (OSP = 0% vs OSP > 0%) was evaluated with Fisher’s exact test, per tissue type and sub-cellular location. To take into account the paired-tissue design of this TMA, we further evaluated DDR1 relative expression, which was categorized in three expression patterns: Equal expression (cancerous OSP = adjacent benign OSP), higher expression (cancerous OSP > adjacent benign OSP), and lower expression (cancerous OSP < adjacent benign OSP). The association between high/low grade GS and DDR1 relative expression patterns was then evaluated using Fisher’s exact test, per subcellular location.

The association between DDR1 and GS was further evaluated with multivariate logistic regression adjusted for tumor/node/metastasis (TNM) staging, classified as local (T0N0MX, T2N0MX, T2NXMX, and T2XN0MX) and advanced (T2XN1MX, T3AN0MX, T3AN1MX, T3ANXMX, T3BN0MX, and T3BN1MX) for each subcellular location. These evaluations were performed with DDR1’s OSP from cancer, OSP from normal, and relative DDR1 relative expression pattern (higher, equal, or lower expression), respectively.

For this cohort of TMA, we obtained de-identified baseline characteristics and clinical outcomes overall survival (OS), biochemical recurrence free survival (BCRFS), and cause-specific death. Associations between DDR1 expression and clinical outcomes such as OS and BCRFS were also performed with the Cox model. Competing risk analysis of cause-specific death was not performed as there were a very low event of death due to PCa in this cohort. All p values are 2-sided with a significance level of 0.05. The results should be regarded only as descriptive findings and multiple testing were not adjusted. All calculations were performed with statistical software R version 3.6.1.

## Results and discussion

**Fig. 1:**
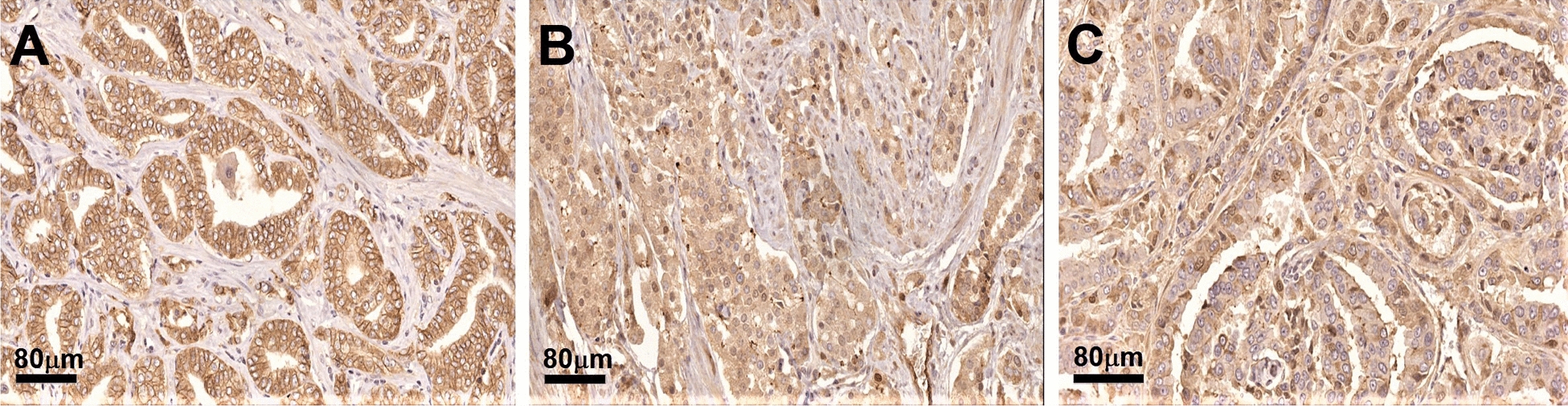
Immunohistochemical localizations of DDR1 in prostate cancer tissues. Representative areas showing membranous (**A**), cytoplasmic (**B**), and predominantly nuclear or perinuclear (**C**) DDR1 immunoreactivity using the Rab‑819 antibody. Note negative staining in stroma surrounding epithelial cells. Images shown were captured at 40× magnifications

**Fig. 2:**
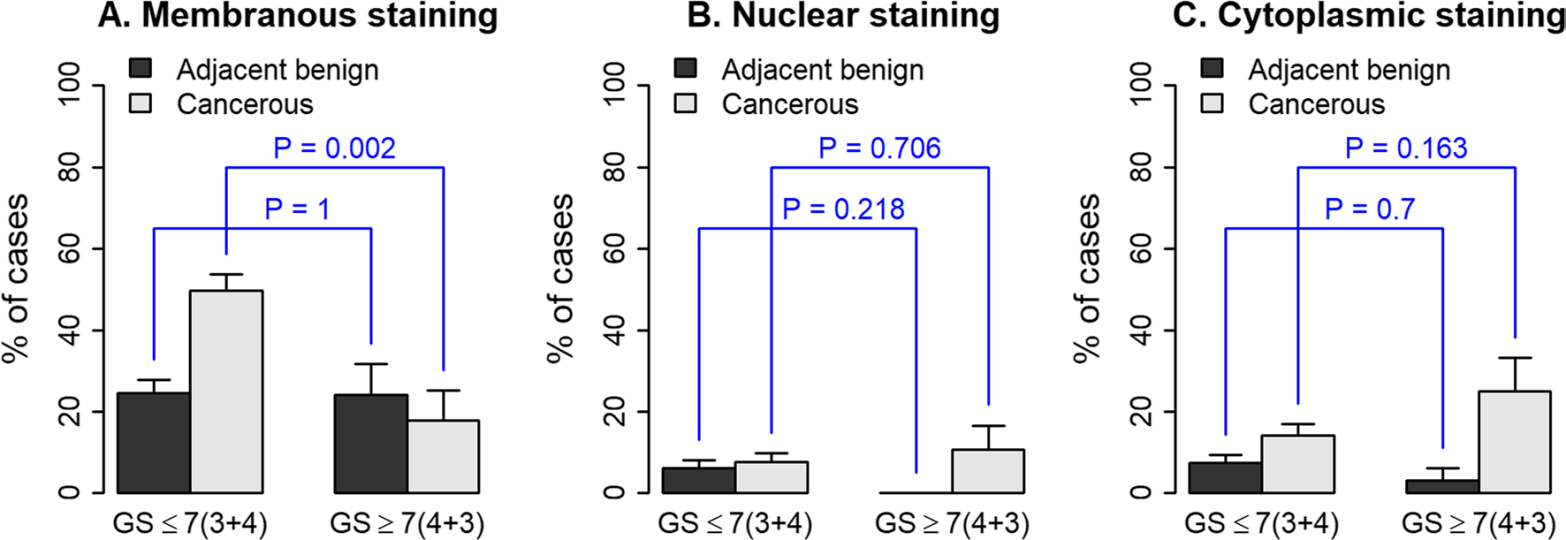
DDR1 expression in different subcellular compartments of cancerous and adjacent benign tissue of low and high GS tumors. DDR1 immunostaining in membrane (**A**), nucleus (**B**), and cytoplasm (**C**) of tumor areas corresponding to low (≤ 7 [3 + 4]) and high (≥ 7 [4 + 3]) GS scores. Levels of DDR1 expression in the different subcellular compartments of cancerous areas were defined as positive (OSP > 0%) or negative (OSP = 0%), as described in the Methods section. Only positive samples are displayed. The association between DDR1 expression and GS category was tested using Fisher’s exact test in each tissue type, cancerous or adjacent benign, respectively. *p* values lower than 0.05 denote statistically significant differences

**Fig. 3:**
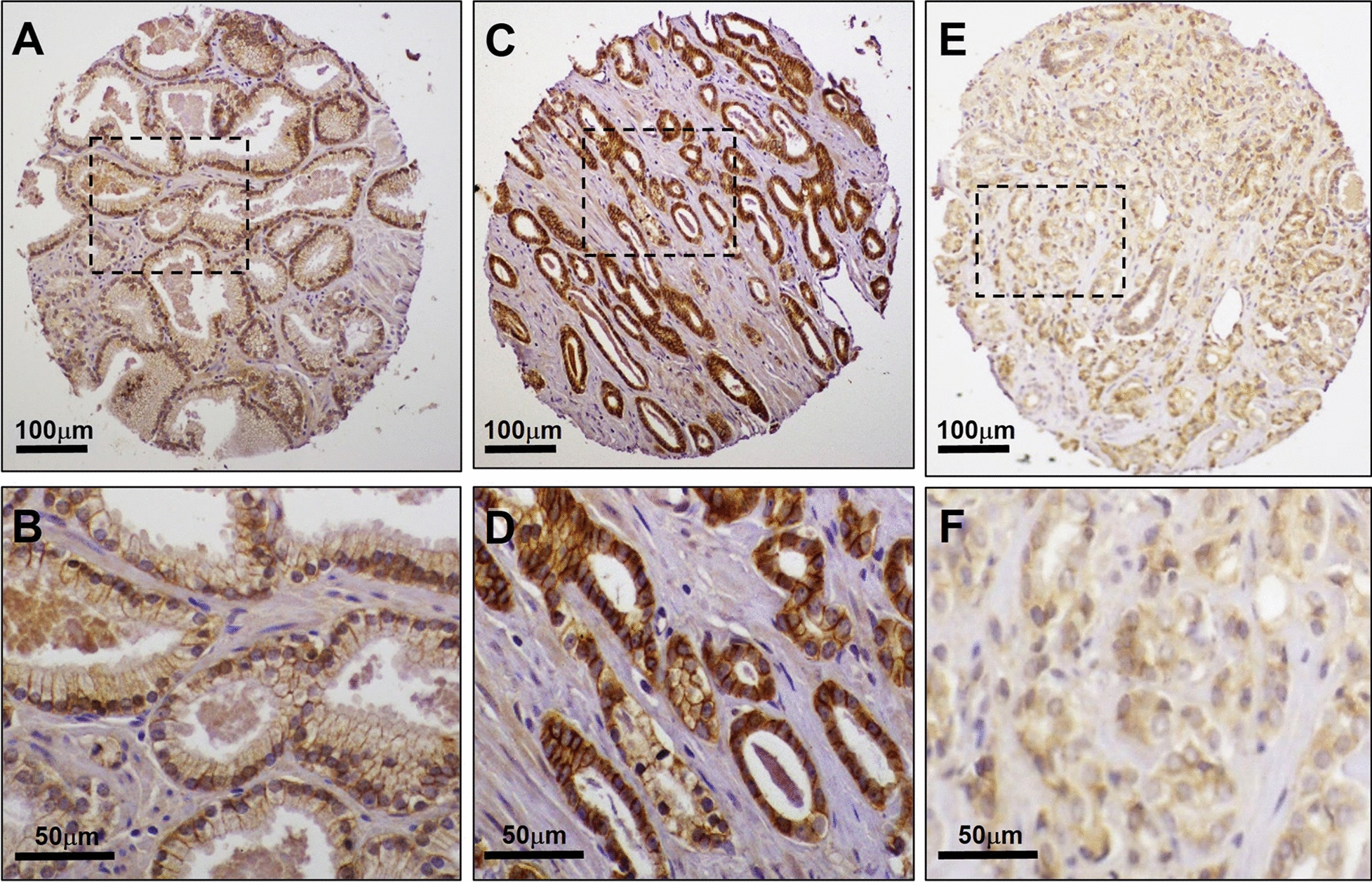
Immunohistochemical staining of DDR1 in cancer tissue microarray cores obtained from radical prostatectomy specimens. Representative image of a core containing normal prostate glands that display weak basolateral DDR1 protein expression (**A**), with outlined area shown at higher magnification (**B**) using the Rab‑819 antibody. GS 6 (3 + 3) lesions with strong membranous DDR1 immunostaining (**C**, **D**). High grade lesion (GS 9 [4 + 5]) with weak cytoplasmic but non-detectable membranous DDR1 staining (**E**, **F**)

Using a TMA containing 1600 cores derived from radical prostatectomies from patients diagnosed with PCa, we examined the association between DDR1 expression through IHC and disease aggressiveness as defined by GS. The demographics and clinicopathologic features associated with the TMA cohort used for this study are shown in Table 1. Figure 1 shows representative IHC patterns detected by the Rab-819 antibody. Note the membranous (Fig. 1A) and nuclear (Fig. 1C) staining of DDR1. Because the Rab-819 antibody not only identified DDR1 at the cell membrane but also in the cytoplasm and/or the nucleus (Fig. 1B), we set to evaluate the tissue specimens for the subcellular distribution of DDR1 as a function of malignancy. For adjacent normal tissues there was no association between DDR1 expression and GS in any of the three subcellular locations examined (Fig. 2). In contrast, for cancer tissues, the fraction of cases that displayed membranous DDR1 expression was significantly lower in high GS tumors than in low GS tumors (Fig. 2A; p = 0.002). While these analyses suggest that DDR1 membranous expression decreases as tumors become more aggressive (high GS), this difference in membranous DDR1 staining between high and low GS cancerous areas did not translate into differences in nuclear or cytoplasmic DDR1 localization between low and high GS tumors (Fig. 2B, C; p = 0.71 and 0.16, respectively). Thus, the decrease in membranous DDR1 in more aggressive PCa tumors was not accompanied by a measurable change in DDR1 positivity in the other two subcellular locations, as indicated. Representative photographs of these results are shown in Fig. 3. Although DDR1 protein expression can be seen in the cell membrane of some benign glands (Fig. 3A, B), there is a trend towards enhanced membranous DDR1 expression in cancerous glands of lower GS cancers (Fig. 3C, D), which is less frequent in cancerous cells of higher GS tumors (Fig. 3E, F).

### Membranous DDR1 expression in cancer vs. normal matched tissue is associated with low GS

**Fig. 4:**
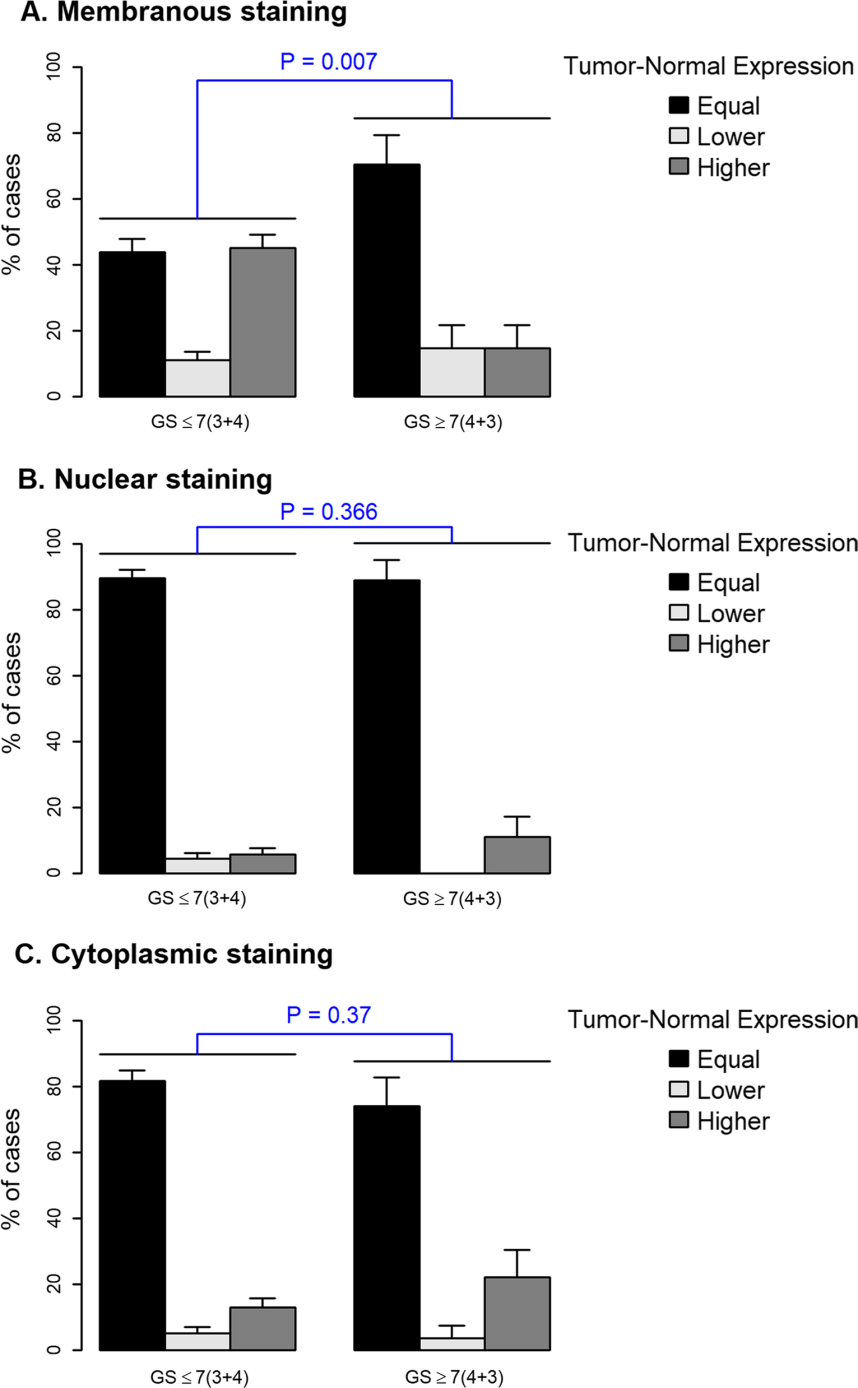
Comparison of membranous, cytoplasmic, and nuclear DDR1 expressions in tumor areas relative to those in corresponding adjacent benign tissue as a function of low and high GS. DDR1 immunostaining in membrane (**A**), nucleus (**B**) and cytoplasm (**C**) of tumor areas corresponding to low (≤ 7 [3 + 4]) and high (≥ 7 [4 + 3]) GS score. Relative DDR1 was categorized as equal expression (cancerous OSP = adjacent benign OSP), higher expression (cancerous OSP > adjacent benign OSP), and lower expression (cancerous OSP < adjacent benign OSP). Statistical comparisons were made using Fisher’s exact test. *p* values lower than 0.05 denote statistically significant differences

Next, we compared membranous, cytoplasmic, and nuclear DDR1 expression in tumor tissues relative to that detected in corresponding adjacent normal tissues, in high and low GS tumors. As shown in Fig. 4A, these analyses revealed that the percentage of cases with low grade GS tumors with DDR1 immunoreactivity at the membrane was significantly higher than that observed in the high GS tumors (Fisher test, p = 0.007). In contrast, no significant differences in relative DDR1 expression at nuclear or cytoplasmic locations were found between low and high GS tumors (Fig. 4B, C). These data indicate that membranous expression of DDR1 is reduced in high GS tumors when compared to low GS tumors, and thus this subcellular location establishes an association between DDR1 and GS in PCa tumors. A previous study by Shimada et al*.* failed to find an association between DDR1 expression and GS in PCa [39]. The reason for this discrepancy is likely to be due to the epitope recognized by the antibodies used for IHC. Shimada et al. [39] utilized an Ab to DDR1 raised against its C-terminal region (intracellular), whereas the Rab-819 antibody recognizes the extracellular domain of DDR1. The reason for the reduced immunoreactivity of DDR1 in the membrane of the high GS tumors reported here remains unknown. However, it could be due to posttranslational receptor regulation, which may include enhanced receptor endocytosis [43–45] and/or cleavage [46–48], all of which may decrease the levels of DDR1 at the cell surface and, consequently, Rab-819 Ab immunoreactivity. The differences in the levels of membranous DDR1 between low and high GS tumors may also be related to the levels of collagen IV in the basement membranes of the PCa tumors. It is well known that collagen IV is a major DDR1 ligand [2], and thus binding of collagen IV to DDR1 may play a role in mediating the interactions of prostate epithelial cells with their underlying basement membrane. Evidence has shown that in the course of PCa progression there is a marked loss of basement membrane and collagen IV [49, 50]. It is therefore tempting to speculate that the decreased expression of membranous DDR1 in high GS tumors represents a response to the loss of basement integrity as tumors become more aggressive.

### DDR1 localizes in the nucleus of cultured prostate epithelial cells

**Fig. 5:**
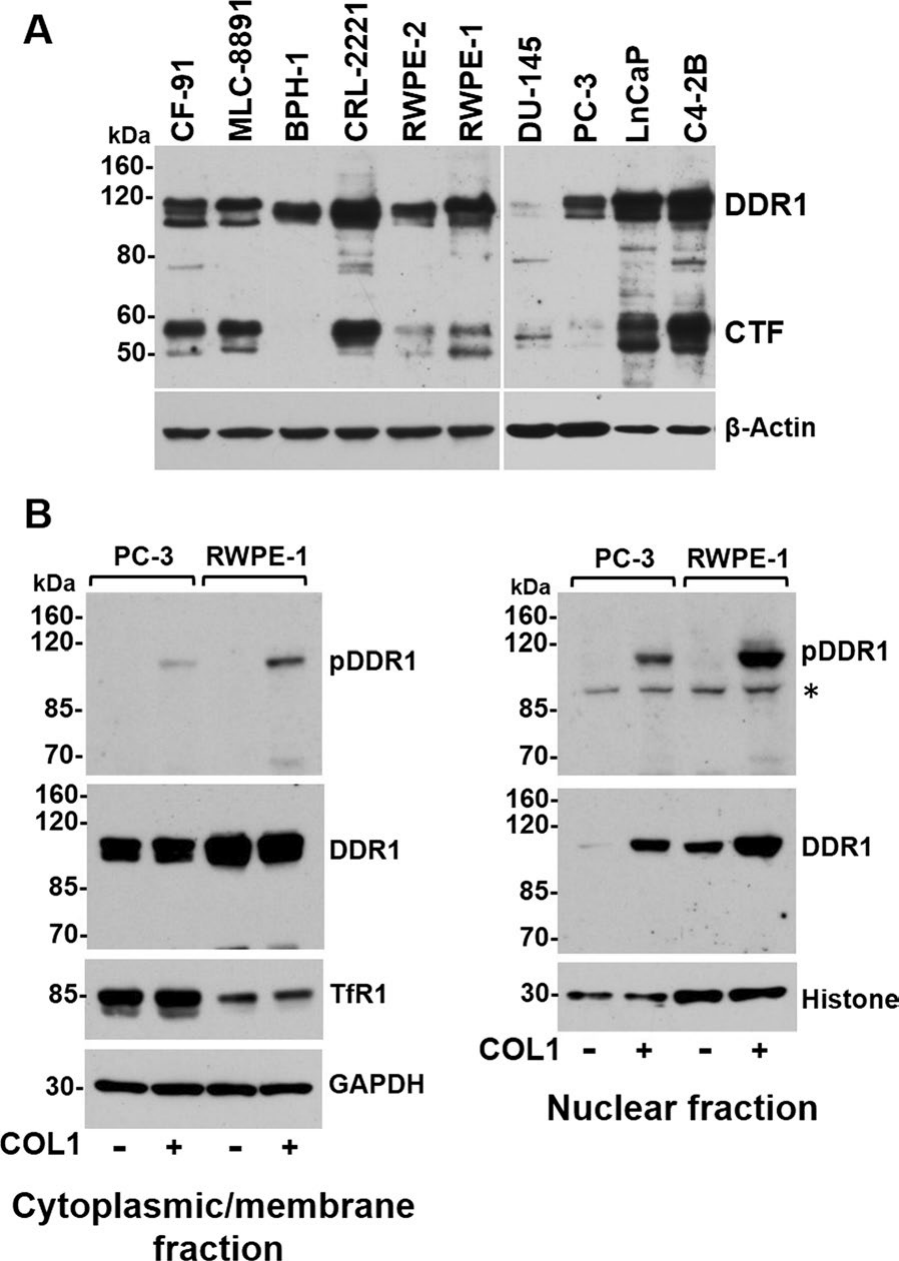
Expression and localization of DDR1 in prostate epithelial cell lines. **A** Human prostate epithelial cell lines were lysed and resolved by reducing SDS-PAGE followed by immunoblot analyses with anti-DDR1 Sc-532 antibody. CTF: C-terminal fragment of DDR1. **B** PC-3 malignant and RWPE-1 non-malignant prostate epithelial cells were treated for 90 min with collagen type I, as described in Materials and Methods. The cells were then processed for subcellular fractionation to obtain the cytoplasmic/membrane (**B**, left panel) and the nuclear (**B**, right panel) fractions, which were then resolved by reducing SDS-PAGE followed by immunoblot analyses with antibodies D1G6 and E1N8F against total DDR1 and phosphorylated DDR1 (pDDR1), respectively, as described in the Methods section. The blots containing the cytoplasmic/membrane fraction were reprobed with Abs to GAPDH (cytoplasmic) and transferrin receptor1 (TfR1, membrane anchored) whereas the nuclear fraction blots were reprobed with Abs to histone, as controls

Our IHC analyses revealed that a relatively higher DDR1 immunoreactivity was evident in the nuclei of PCa cells in high GS tumors than in those of low GS tumors (11 vs. 6%, respectively, Fig. 4B), suggesting that a subgroup of high GS tumors displays areas harboring cells with elevated nuclear DDR1 expression. Considering that DDR1 is a type I transmembrane protein, the presence of DDR1 in the nucleus is noteworthy. It is well established that several RTKs can translocate to the nucleus, where they can regulate gene expression [51, 52]. Importantly, a recent study by the Pozzi laboratory demonstrated that DDR1 can also be detected in the nucleus [53]. Based on theirs results and our findings in the tissue specimens, we wished to examine whether DDR1 can be detected in the nuclear fraction of cultured human prostate epithelial cell lines. To this end, we first examined established prostate cell lines for expression of DDR1 in whole cell lysates under steady state conditions by immunoblot analyses. As shown in Fig. 5A, all non-malignant prostate cell lines (CF-91, MLC-8891, BPH-1, CRL-2221, RWPE-2 and RWPE-1) express DDR1 (~ 120 kDa). As for the human PCa cell lines studied herein (DU-145, PC-3, LNCaP and C4-2B), all express DDR1, except for DU145 cells. The lysates also contained a specific immunoreactive ~ 57-kDa protein representing proteolytically cleaved DDR1, which lacks the entire ectodomain and comprises a membrane-anchored C-terminal fragment (CTF) [47, 48]. Next, we examined the subcellular distribution (cytoplasmic/membrane, and nuclear) of DDR1 in untreated and collagen I-treated malignant PC-3 cells and immortalized non-malignant RWPE-1 prostate epithelial cells. As expected, total DDR1 receptor was detected in the cytoplasmic/membrane fractions, which was phosphorylated in response to collagen treatment in both cell lines (Fig. 5B, left panel). These analyses also revealed that total DDR1 could also be detected in the nuclear fraction of both PC-3 and RWPE-1 cells, with PC-3 cells showing relatively lower levels than RWPE-1 cells, under these conditions (Fig. 5B, right panel). Upon collagen stimulation, phosphorylated DDR1 was clearly detected in the nuclear fraction of both cell lines, with RWPE-1 cells showing relatively higher levels of nuclear phosphorylated DDR1. Because collagen is expected to bind and activate DDR1 at the cell surface, these results suggest that collagen-evoked receptor activation is followed by translocation of membrane-bound DDR1 to the nucleus. These results support the findings of nuclear DDR1 in our IHC studies and agree with the recent studies of Chiusa et al*.* [53]. Collectively, these results add DDR1 to the list of RTK family members endowed with the ability to translocate to the nucleus [51, 52]. At present, the role of nuclear DDR1 in benign and cancerous prostate epithelial cells and its association with disease progression remain to be elucidated.

### Membranous DDR1 as a predictor of aggressiveness and association with clinical outcome

The demographics and clinicopathologic features associated with the TMA cohort used for this study are shown in Table 1. We found that neither cytoplasmic nor nuclear DDR1 showed predictive value (data not shown) from our multivariate logistic regression analyses. However, while there was no significant association between DDR1 membranous expression and GS in adjacent normal tissue, consistent with the univariate analysis, DDR1 membranous expression in cancerous tissue is an independent predictor for tumor aggressiveness with statistically significant odds ratio of 0.97 (p = 0.001) (Table 2). For each 1% increase of OSP, the odds of having high GS are lowered 3%. Thus, the difference in DDR1 membranous expression in cancerous vs. adjacent benign tissue is an independent predictor of tumor aggressiveness. Because the event of OS or BCRFS were low in this cohort, only the results from the univariate Cox model analysis for OS or BCRFS are shown (Additional file 2: Table S2). Whereas traditional predictors, such as age, TNM, and Gleason scores, are significant predictors of OS and BCRFS, DDR1 expression is not.

**Table 2:** Multivariate logistic regression of tumor aggressiveness of DDR1 membranous immunostaining percentage adjusted for TNM stage

	Cancerous tissues^a^		Adjacent Normal tissues^a^		Paired cancerous—normal^b^	
	OR (95% CI)	p value	OR (95% CI)	p value	OR (95% CI)	p value
DDR1	0.97 (0.95, 0.99)	0.001	1.00 (0.98, 1.03)	0.727	0.97 (0.96,0.99)	0.001
TNM stage advanced (ref: local)	8.82 (3.39, 22.94)	< 0.001	11.61 (4.65, 28.96)	< 0.001	11.44 (4.16, 31.48)	< 0.001

OR: odds ratio, CI: confidence interval

^a^DDR1 staining intensity OSP

^b^DDR1 staining intensity difference (OSP in cancerous tissue minus OSP in normal tissue)

In summary, this study evaluated the expression of DDR1 in a large cohort of PCa samples by IHC. We focused our analyses in evaluating DDR1 at various subcellular localizations that display positive immunoreactivity, which clearly identified DDR1 in the plasma membrane, the cytoplasm, and the nucleus. Thus, in this regard, this study is the first to evaluate the association between DDR1 subcellular localization and GS in PCa samples. Our data suggest that membrane, but not cytoplasmic or nuclear, localization of DDR1 better reflects the aggressiveness of PCa as defined by GS. We found that reduced positivity of DDR1 at the plasma membrane defines tumor lesions with high GS. Conversely, low GS cancers are characterized by higher levels of DDR1 at the plasma membrane. Therefore, our study highlights the notion that associations between cancer malignancy and DDR1 expression at least in PCa may need to address receptor subcellular location. Thus, based on our observations on DDR1 distribution, we surmise that membranous DDR1 is likely to play a role in the early stages of PCa development by mediating the interactions with the ECM. Consistent with the importance of DDR1-matrix interactions, our data showed that, overall, membranous DDR1 localization was a strong predictor of PCa aggressiveness. Finally, we showed that DDR1 is present in the nuclear compartment of PCa cells both in tissues and in cultured cell lines, reflecting a potential role for DDR1 in tumor cell behavior through receptor activity within the nuclear environment. More studies are warranted to define the mechanisms that regulate DDR1 traffic to the nucleus and its role in PCa progression.

## Supplementary Information

**Additional file 1: Table S1.** Clinical outcome variables.

**Additional file 2: Table S2.** Results of Univariate Cox model for OS and BCRFS.

## Data Availability

All the data used to support the findings of this study are included within the article and its supplementary information files. Please contact author for data requests.

## Abbreviations

ATCC: American Type Culture Collection
BCR: Biochemical recurrence
BCRFS: Biochemical recurrence free survival
BPH: Benign prostatic hyperplasia
CTF: C-terminal fragment
DDR: Discoidin Domain Receptor
DS: Discoidin domain
FBS: Fetal bovine serum
GAPDH: Glyceraldehyde 3-phosphate dehydrogenase
GS: Gleason score
IHC: Immunohistochemistry
mAb: Monoclonal antibody
OST: Overall staining percentage
PBS: Phosphate-buffered saline
PCa: Prostate cancer
PCBN: Prostate Cancer Biorepository Network
PSA: Prostate-specific antigen
RP: Radical prostatectomy
RT: Radiation therapy
RTK: Receptor tyrosine kinase
TMA: Tissue microarray
TNM: Tumor/node/metastasis
WSU: Wayne State University
