# Supplementary material for: Expression and subcellular localization of Discoidin Domain Receptor 1 (DDR1) define prostate cancer aggressiveness
Source: Cancer Cell Int. 2021 Sep 21;21:507. doi: 10.1186/s12935-021-02206-1 (PMC8456559; doi:10.1186/s12935-021-02206-1)
Supplement: Supplementary file 1 — Additional file 1: Table S1. Clinical outcome variables. [file 12935_2021_2206_MOESM1_ESM.docx]

**Table S1**. **Clinical outcome variables.**

|  | Biochemical  Recurrence | Death due to  any cause | Death due to  Prostate Cancer |
| --- | --- | --- | --- |
| NO | 148 | 158 | 176 |
| YES | 24 | 21 | 3 |
| Missing | 28 | 21 | 21 |
