# Supplementary material for: Expression and subcellular localization of Discoidin Domain Receptor 1 (DDR1) define prostate cancer aggressiveness
Source: Cancer Cell Int. 2021 Sep 21;21:507. doi: 10.1186/s12935-021-02206-1 (PMC8456559; doi:10.1186/s12935-021-02206-1)
Supplement: Supplementary file 2 — Additional file 2: Table S2. Results of Univariate Cox model for OS and BCRFS. [file 12935_2021_2206_MOESM2_ESM.docx]

**Table S2**. **Results of Univariate Cox model for OS and BCRFS***

|  | **OS** | | |  | **BCRFS** | | |
| --- | --- | --- | --- | --- | --- | --- | --- |
|  | **n** | **HR (95% CI)** | **P value** |  | **n** | **HR (95% CI)** | **P value** |
| **Age** | 179 | 1.13 (1.05-1.21) | ***<0.001*** |  | 178 | 1.08 (1.03-1.13) | ***0.003*** |
| **Body mass index** | 177 | 1.01 (0.88-1.15) | 0.900 |  | 176 | 0.99 (0.90-1.09) | 0.783 |
| **PSA at diagnosis** | 179 | 1.03 (0.96-1.11) | 0.379 |  | 178 | 1.14 (1.08-1.20) | ***<0.001*** |
| **Race** |  |  |  |  |  |  |  |
| Caucasian | 158 | Ref. |  |  | 157 | Ref. |  |
| Other | 21 | 1.86 (0.54-6.34) | 0.322 |  | 21 | 1.41 (0.55-3.63) | 0.471 |
| **Family history of prostate cancer** |  |  |  |  |  |  |  |
| No | 100 | Ref. |  |  | 99 | Ref. |  |
| Yes | 66 | 1.51 (0.61-3.71) | 0.374 |  | 66 | 1.07 (0.56-2.06) | 0.833 |
| **TNM stage** |  |  |  |  |  |  |  |
| Local | 123 | Ref. |  |  | 122 | Ref. |  |
| Advanced | 56 | 2.12 (0.90-4.99) | 0.086 |  | 56 | 4.07 (2.13-7.76) | ***<0.001*** |
| **Gleason score** |  |  |  |  |  |  |  |
| 6 | 100 | Ref. |  |  | 99 | Ref. |  |
| 3+4 | 49 | 1.74 (0.61-4.97) | 0.300 |  | 49 | 1.67 (0.73-3.86) | 0.228 |
| 4+3 | 10 | 3.64 (0.94-14.13) | 0.062 |  | 10 | 5.14 (1.78-14.87) | ***0.003*** |
| 8 | 11 | 2.34 (0.49-11.27) | 0.290 |  | 11 | 5.74 (2.12-15.56) | ***<0.001*** |
| 9-10 | 9 | 3.11 (0.64-15.05) | 0.159 |  | 9 | 14.11 (5.15-38.65) | ***<0.001*** |
| **Extraprostatic extension** |  |  |  |  |  |  |  |
| No | 127 | Ref. |  |  | 126 | Ref. |  |
| Yes | 51 | 2.55 (1.08-6.00) | ***0.032*** |  | 51 | 3.86 (2.05-7.28) | ***<0.001*** |
| **Lymph node metastases** |  |  |  |  |  |  |  |
| No | 175 | Ref. |  |  | 174 | Ref. |  |
| Yes | 2 | 3.28 (0.44-24.54) | 0.248 |  | 2 | 2.10 (0.29-15.36) | 0.465 |
| **Distant metastasis** |  |  |  |  |  |  |  |
| No | 165 | Ref. |  |  | 164 | Ref. |  |
| Yes | 6 | 6.18 (1.69-22.63) | ***0.006*** |  | 6 | 13.41 (5.28-34.06) | ***<0.001*** |
| **Chemotherapy** |  |  |  |  |  |  |  |
| No | 168 | Ref. |  |  | 167 | Ref. |  |
| Yes | 3 | 10.45 (2.24-48.70) | ***0.003*** |  | 3 | 14.12 (4.18-47.67) | ***<0.001*** |
| **Radiation therapy** |  |  |  |  |  |  |  |
| No | 160 | Ref. |  |  | 159 | Ref. |  |
| Yes | 11 | 0.70 (0.09-5.39) | 0.732 |  | 11 | 10.36 (4.76-22.54) | ***<0.001*** |

**Table S2. (Continued)**

|  | **OS** | | |  | **BCRFS** | | |
| --- | --- | --- | --- | --- | --- | --- | --- |
|  | **n** | **HR (95% CI)** | **P value** |  | **n** | **HR (95% CI)** | **P value** |
| **Hormonal therapy** |  |  |  |  |  |  |  |
| No | 160 | Ref. |  |  | 159 | Ref. |  |
| Yes | 12 | 3.62 (1.13-11.57) | ***0.030*** |  | 12 | 28.69 (12.68-64.94) | ***<0.001*** |
| **Surgical margin status** |  |  |  |  |  |  |  |
| Negative | 153 | Ref. |  |  | 152 | Ref. |  |
| Positive | 26 | 1.84 (0.71-4.74) | 0.208 |  | 26 | 2.49 (1.26-4.92) | ***0.009*** |
| **Seminal vesicle involvement** |  |  |  |  |  |  |  |
| No | 165 | Ref. |  |  | 164 | Ref. |  |
| Yes | 14 | 3.02 (1.01-9.02) | ***0.048*** |  | 14 | 5.78 (2.63-12.70) | ***<0.001*** |
| **Membranous DDR1, Adjacent Benign** |  |  |  |  |  |  |  |
| Negative | 133 | Ref. |  |  | 132 | Ref. |  |
| Positive | 42 | 1.56 (0.61-4.04) | 0.355 |  | 42 | 1.57 (0.77-3.19) | 0.210 |
| **Membranous DDR1, Cancerous** |  |  |  |  |  |  |  |
| Negative | 96 | Ref. |  |  | 96 | Ref. |  |
| Positive | 69 | 1.19 (0.47-3.03) | 0.709 |  | 68 | 1.21 (0.61-2.39) | 0.580 |
| **Membranous DDR1, Paired** |  |  |  |  |  |  |  |
| Equal expression | 81 | Ref. |  |  | 81 | Ref. |  |
| Lower expression | 19 | 2.79 (0.84-9.27) | 0.095 |  | 19 | 1.48 (0.54-4.04) | 0.445 |
| Higher expression | 62 | 1.00 (0.35-2.88) | 0.997 |  | 61 | 1.02 (0.48-2.16) | 0.953 |
| **Cytoplasmic DDR1, Adjacent Benign** |  |  |  |  |  |  |  |
| Negative | 164 | Ref. |  |  | 163 | Ref. |  |
| Positive | 11 | 0.50 (0.07-3.72) | 0.497 |  | 11 | 1.61 (0.57-4.54) | 0.372 |
| **Cytoplasmic DDR1, Cancerous** |  |  |  |  |  |  |  |
| Negative | 138 | Ref. |  |  | 137 | Ref. |  |
| Positive | 27 | 1.39 (0.46-4.24) | 0.559 |  | 27 | 1.02 (0.42-2.47) | 0.962 |
| **Cytoplasmic DDR1, Paired** |  |  |  |  |  |  |  |
| Equal expression | 131 | Ref. |  |  | 130 | Ref. |  |
| Lower expression | 7 | 0.85 (0.11-6.50) | 0.875 |  | 7 | 2.87 (0.99-8.30) | 0.052 |
| Higher expression | 24 | 1.58 (0.52-4.86) | 0.422 |  | 24 | 1.34 (0.54-3.29) | 0.526 |
| **Nuclear DDR1, Adjacent Benign** |  |  |  |  |  |  |  |
| Negative | 165 | Ref. |  |  | 164 | Ref. |  |
| Positive | 10 | 0.00 (0.00-Inf) | 0.998 |  | 10 | 0.92 (0.22-3.83) | 0.907 |
| **Nuclear DDR1, Cancerous** |  |  |  |  |  |  |  |
| Negative | 151 | Ref. |  |  | 150 | Ref. |  |
| Positive | 14 | 0.58 (0.08-4.37) | 0.598 |  | 14 | 1.39 (0.49-3.96) | 0.534 |
| **Nuclear DDR1, Paired** |  |  |  |  |  |  |  |
| Equal expression | 144 | Ref. |  |  | 143 | Ref. |  |
| Lower expression | 7 | 0.00 (0.00-Inf) | 0.998 |  | 7 | 1.30 (0.31-5.46) | 0.723 |
| Higher expression | 11 | 0.62 (0.08-4.64) | 0.640 |  | 11 | 1.07 (0.32-3.51) | 0.916 |

***Note:** sample sizes are smaller than those shown in Table 1 due to missing OS or BCRFS values.
